# Aroma formation during cheese ripening is best resembled by *Lactococcus lactis* retentostat cultures

**DOI:** 10.1186/s12934-018-0950-7

**Published:** 2018-07-04

**Authors:** Oscar van Mastrigt, Diego Gallegos Tejeda, Mette N. Kristensen, Tjakko Abee, Eddy J. Smid

**Affiliations:** 1 Food Microbiology, Wageningen University & Research, P.O. Box 17, 6700AA Wageningen, The Netherlands; 2 Arla Innovation Centre, Arla Foods Amba, Agro Food Park 19, 8200 Aarhus N, Denmark

**Keywords:** Fermentation, VOC, Zero growth, Metabolism

## Abstract

**Background:**

Cheese ripening is a complex, time consuming and expensive process, which involves the generation of precursors from carbohydrates, proteins and fats and their subsequent conversion into a wide range of compounds responsible for the flavour and texture of the cheese. This study aims to investigate production of cheese aroma compounds outside the cheese matrix that could be applied for instance as food supplements in dairy or non-dairy products.

**Results:**

In this study, aroma formation by a dairy *Lactococcus lactis* was analysed as a function of the growth medium [milk, hydrolysed micellar casein isolate (MCI) and chemically defined medium (CDM)] and the cultivation conditions (batch culture, retentostat culture and a milli-cheese model system). In the retentostat cultures, the nutrient supply was severely restricted resulting in low growth rates (~ 0.001 h^−1^), thereby mimicking cheese ripening conditions in which nutrients are scarce and bacteria hardly grow. In total 82 volatile organic compounds were produced by the bacteria. Despite the use of a chemically defined medium, retentostat cultures had the biggest qualitative overlap in aroma production with the milli-cheese model system (36 out of 54 compounds). In the retentostat cultures, 52 known cheese compounds were produced and several important cheese aroma compounds and/or compounds with a buttery or cheese-like aroma increased in retentostat cultures compared to batch cultures and milli-cheeses, such as esters, methyl ketones, diketones and unsaturated ketones. In cultures on CDM and MCI, free fatty acids and their corresponding degradation products were underrepresented compared to what was found in the milli-cheeses. Addition of a mixture of free fatty acids to CDM and MCI could help to enhance flavour formation in these media, thereby even better resembling flavour formation in cheese.

**Conclusions:**

This study demonstrates that retentostat cultivation is the preferred method to produce cheese flavours outside the cheese matrix by mimicking the slow growth of bacteria during cheese ripening.

**Electronic supplementary material:**

The online version of this article (10.1186/s12934-018-0950-7) contains supplementary material, which is available to authorized users.

## Background

Ripening of cheese is a complex process in which the typical cheese characteristics, such as the flavour and texture, are formed by the action of numerous enzymes derived from the milk, the rennet, the starter bacteria and the non-starter bacteria [1]. It primarily involves the generation of precursors from carbohydrates, proteins and fats and their conversion into a wide range of compounds responsible for the flavour and texture of the cheese [2].

Cheese ripening is a slow and expensive process that can last from about 2 weeks, e.g. mozzarella, to more than 2 years, e.g. Parmigiano-Reggiano [3]. Consequently, there is a demand to accelerate this complex process while maintaining the flavour balance. Most of the methods to accelerate cheese ripening focus on enhancing proteolysis and lipolysis by for instance ripening at elevated temperatures, the addition of enzymes (e.g. lipases, proteinases and peptidases), microencapsulation of enzymes, using attenuated starter cultures, culture adjuncts and genetically modified cultures. Each of these methods have their advantages and disadvantages that are extensively reviewed by Azarnia et al. [4], but have in common that they mainly enhance precursor formation and not their subsequent conversion into aroma compounds which requires intact cells with functional metabolic pathways [2].

From both a scientific and a technological perspective, it could be interesting to study aroma formation outside the cheese matrix. Such a study could provide new insights into for instance the origin of particular aroma compounds and how their formation is regulated. Moreover, such studies could help to optimise or steer aroma formation by lactic acid bacteria in such a way that these aroma compounds can be applied as food supplements in dairy or dairy-like products.

It is important to realise that during the ripening of cheese the bacteria hardly grow, and this has been shown to affect the aroma formation [5, 6]. Slow growth in the cheese matrix could be mimicked using retentostat cultivation, which is a modification of the chemostat cultivation in which the biomass is retained in the bioreactor using a filter in the effluent line [7]. However, milk cannot be used as medium in retentostat cultures due to clogging of the filter. Therefore, retentostat cultivations were carried out using a chemically defined medium with lactose, citrate and Bacto-tryptone as main carbon and nitrogen sources to mimic the composition of milk as much as possible within the constraints imposed by the retentostat cultivation method.

This study aimed at comparing aroma formation by the dairy *Lactococcus lactis* subsp. *lactis* biovar diacetylactis FM03-V1 during (i) growth in retentostat cultures using chemically defined media, (ii) in batch cultures using chemically defined media, hydrolysed micellar casein isolate (MCI) and milk, and (iii) in a milli-cheese model system [8].

## Methods

### Strain and pre-culture conditions

*Lactococcus lactis* subsp. *lactis* biovar *diacetylactis* FM03-V1 [9, 10], which has been isolated from a 10-week-old Samsø cheese, was used in this study. For each cultivation, *L. lactis* was streaked on M17 plates (Oxoid, UK) supplemented with 0.5% (w/v) lactose (LM17) and incubated for 48 h at 30 °C. A single colony was inoculated into appropriate liquid medium (chemically defined medium, hydrolysed MCI or milk) and incubated overnight at 30 °C. These overnight cultures were used to prepare the milli-cheeses and to inoculate the same media for the batch and retentostat cultivations.

### Media

#### Chemically defined medium (CDM)

The chemically defined media contained 0.5% (w/w) lactose, 0.24% (w/w) (NH_4_)_3_citrate and 1% (w/w) Bacto-Tryptone as main carbon and nitrogen sources [9] and the pH was standardized to 5.5 with HCl. To increase the buffering capacity the phosphate content was increased from 2.67 to 6 g/kg KH_2_PO_4_ and from 0 to 6 g/kg K_2_HPO_4_ for batch cultivations.

#### Hydrolysed micellar casein isolate

Micellar casein isolate (MCI) powder was obtained from Arla Foods (Aarhus, Denmark) and stored as powder at 4 °C until use. The MCI powder was dissolved in demineralised water to a protein content of 5.2%. Subsequently, the solution was heated to 92 °C for 45 min and cooled down to 50 °C. The pH was adjusted to 8 with 6 M NaOH and 0.018% Alcalase^®^ 2.4 L FG (Novozymes, Denmark) was added. After incubation at 50 °C until the pH reached 7, a solution of 6 M NaOH was used to adjust the pH to 7 if required and 0.018% Neutrase 0.8 L (Novozymes, Denmark) was added. After incubation for 4 h, enzymes were inactivated at 85 °C for 15 min. Subsequently, 0.027% Flavourzymes 1000 L (Novozymes, Denmark) was added and the MCI solution was incubated for 18 h at 50 °C. Finally, enzymes were inactivated at 85 °C for 15 min. After centrifugation for 1 h at 10 °C at 15,600×*g*, the hydrolysed MCI was sterilised by filtration through a 0.2 µm filter (Millipore, USA).

### Cultivations

#### Batch cultures

*Lactococcus lactis* was incubated in at least 5 independent batch cultures at 30 °C for 2 weeks. Three types of medium were used: full fat UHT milk, hydrolysed MCI and chemically defined medium. For the milk pre-cultures, skimmed UHT milk supplemented with 1% (w/w) Bacto-tryptone was used.

#### Retentostat cultures

Two independent retentostat cultivations were performed in bioreactors with a 1 L working volume (Infors HT, Switzerland). The stirring speed was set at 400 rpm, the pH was controlled at 5.5 by automatic addition of 5 M NaOH and the temperature was kept at 30 °C. The headspace was flushed with nitrogen gas at a flow of 0.1 L/min to maintain anaerobic conditions. After running the bioreactor for at least five volume changes in a chemostat mode at a dilution rate of 0.05 h^−1^, a polyethersulfone crossflow filter (Spectrum laboratories, USA) was connected to the effluent line in an outer loop to start the retentostat cultivation. Samples were taken every 3–4 days, and the aroma profile was compared with the other cultures after 2 weeks.

#### Milli-cheese

Milli-cheeses were made with pasteurised full-fat milk (Jumbo, Netherlands) according to Spus et al. [8] with some adaptations. 1% (w/w) Bacto-tryptone was added to the milk so no caseinolytic strain had to be added. Forty-five millilitres milk was pre-heated at 30.5 °C and 5 mL 10% Bacto-tryptone, 12.5 μL rennet, 20 μL of 33% (w/v) CaCl_2_ and 0.5 mL overnight culture were added and mixed. A 24-deep well plate was filled with 5 mL of this mixture per well and incubated at 32 °C for 40 min on a thermoblock (Eppendorf, USA). Subsequently, the curd was cut with a custom-made sterile stirring device for 20 min (stirring for 20 s, resting for 3 min) followed by 5 min of resting. The plate was sealed with an adhesive cover (Microseal^®^, Bio-Rad, USA) and centrifuged at 500×*g* for 30 min at 30 °C. The whey was removed and replaced by 2 mL sterile water pre-heated at 45 °C. The curd was cut for 40 min at 35 °C and rested for 20 min. The plate was sealed again and centrifuged for 1 h at 4800×*g* at 30 °C. After all the whey was removed, the plate was sealed with a gas-permeable seal (BREATHseal™, Greinier Bio One, Germany) and incubated at 30 °C. After overnight incubation, 50 μL of a 19% (w/v) NaCl solution was added to each well followed by centrifugation for 5 min at 99×*g*. The plate was sealed with a gas-permeable seal, placed in a jar under anaerobic conditions and incubated at 12 °C. After 1, 2, 4 and 8 weeks, 6 cheeses per time point were sampled for analysis of the volatile organic compounds.

### Cell dry weight determination

The cell dry weight was determined as described by van Mastrigt et al. [9]. Approximately 3 mL sample was used for each determination.

### Cell viability detection

The viability of cells in the culture was determined by LIVE/DEAD *Bac*light Bacterial Viability kit (Molecular Probes Europe, The Netherlands). Cells were stained by incubating 100 μL culture with 3.34 μM green fluorescent SYTO9 and 20 μM red fluorescent propidium iodide for 10 min at room temperature in the dark. The number of green and red cells were counted after visualising them with an X-Cite 120Q excitation light source (Excelitas, USA) at 1000 times magnification with a fluorescent microscope equipped with a camera (Olympus, Japan). Selected images for counting had at least 50 cells.

### Volatile organic compounds (VOCs) analysis

One millilitre sample or approximately 0.5 g cheese was transferred to a 5 mL GC vial and stored at − 20 °C until analysis of the VOCs by headspace solid phase microextraction gas chromatography mass spectrometry (HS SPME GC–MS) according to van Mastrigt et al. [6]. Aroma profiles were analysed with Chromeleon 7.2 software. The ICIS algorithm was used for peak integration and mass spectral profiles were matched with the NIST main library for identification. One peak (in general the higher m/z peak per compound) was used per compound for quantification and 1 or 2 peaks were used for confirmation. Compounds were only considered if the area of the quantitative peak was at least three times higher than the peak area in the medium in at least one sample and the peak area was higher than 500 count × min. Methyl esters, butyl esters and 1-butanol were removed from the analysis as their formation was dependent on the presence of methanol and butanol, which were not produced by the bacteria but present in the medium (1-butanol is used as solvent to make Bacto-tryptone). Alkanes were removed because they were not considered as important flavour compounds in cheese. Compounds were considered produced if the peak area was at least 1.5 times higher than the peak area in the medium and the peak area was higher than 200 count × min. The aroma profiles of every biological independent culture was analysed once, except for the two retentostat cultures, which were analysed in duplicate.

## Results and discussion

*Lactococcus lactis* FM03-V1 was grown in retentostat and batch cultures on a chemically defined medium (CDM) to determine the effect of the cultivation method, imposing slow and fast growth respectively, on the formation of aroma compounds. Furthermore, aroma formation was analysed in batch cultures using three different media [CDM, hydrolysed micellar casein isolate (MCI) and full fat milk] and in a milli-cheese model system, which could indicate the origin of the compounds and show which methods best resemble aroma formation during cheese ripening. We chose to use a milli-cheese model system instead of conventionally produced cheese making for several reasons: (i) we wanted to use the single non-proteolytic strain *L. lactis* FM03-V1, which required the addition of an amino acid source to the milk; (ii) milli-cheeses resemble conventionally produced cheeses very well including their flavour profile [11]; (iii) the milli-cheese system allowed for more replicates and different sampling times.

In batch and retentostat cultures (liquid medium) *L. lactis* was incubated for 2 weeks, while aroma formation in the milli-cheeses was analysed after 1, 2, 4 and 8 weeks of ripening. During the retentostat cultivations, the biomass increased in 2 weeks from 1 to 6 gDW/kg and the growth rate gradually decreased from 0.05 h^−1^ to approximately 0.001 h^−1^, while the viability remained always above 90% as determined by live/dead staining (Additional file 1: Figure S1). In the batch cultivations, the bacteria grew fast in the first day after which the growth rate decreased due to nutrient depletion and/or low pH.

### Qualitative comparison of aroma production

**Fig. 1:**
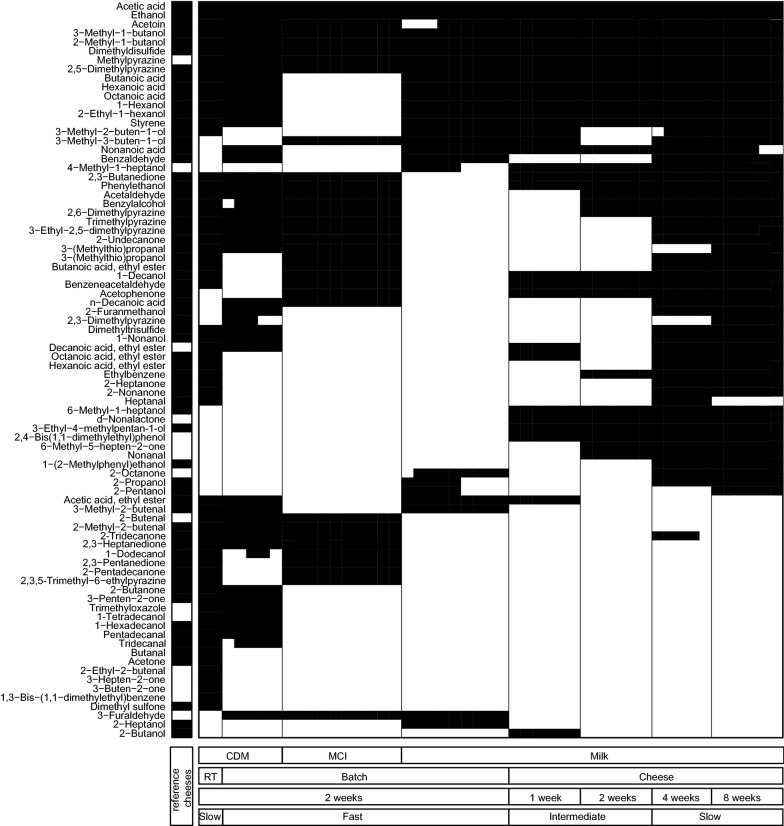
Production of VOCs by *L. lactis* FM03-V1. Colours represent the presence (black) or absence (white) of VOCs after 2 weeks incubation in batch cultures in chemically defined medium (CDM), hydrolysed micellar casein isolate (MCI), and full fat milk, or in retentostat cultures (RT) in CDM or in a milli-cheese model system after 1, 2, 4 or 8 weeks or ripening. The first column represents compounds that can be found in various cheeses (black: present; [13–18])

In total 82 aroma compounds were considered produced in the cultivations (in at least 1 sample at least a three times increase after fermentation and a peak area greater than 500 count × min). The produced compounds were qualitatively compared with each other in a heatmap showing production of compounds in specific cultures (Fig. 1). Biological replicates had highly similar patterns in the heat map showing that aroma formation was reproducible. Furthermore, we found that both the cultivation method (batch cultivation, retentostat cultivation and milli-cheese) and the medium (CDM, MCI, milk) affected the formation of aroma compounds.

Fatty acids were not produced in the MCI media possibly due to the lack of fat in this medium. Interestingly, fatty acids were produced in CDM that also did not contain fat or free fatty acids indicating that free fatty acid were synthesised by the bacteria to end up in volatile aroma compounds. In the batch cultures with milk several degradation products of amino acids were not found while being found in batch culture based on CDM and/or MCI, e.g. phenylethanol, benzeneacetaldehyde, benzylalcohol, acetophenone, 3-(methylthio)propanol, 3-(methylthio)propanal and dimethyltrisulfide. In CDM and MCI, amino acids were abundant due to addition of Bacto-tryptone and due to hydrolysis of the proteins, respectively. There were 10 compounds that were lacking in the batch cultures with CDM and milk while being present in the 8-week-old cheeses and retentostat cultures, which suggests that these compounds were only produced by slow growing cells. These compounds include ethyl esters (butanoic acid, ethyl ester, hexanoic acid, ethyl ester and octanoic acid, ethyl ester) and methyl ketones (2-heptanone and 2-nonanone). The limiting compound in ethyl ester formation by lactic acid bacteria is considered to be ethanol [12]. Notably, due to the low growth rate in retentostat cultures, *L. lactis* produces mainly ethanol, acetate and formate instead of lactate, thereby driving ethyl ester formation. Both esters and methyl ketones strongly contribute to the flavour of the cheese due to their low perception thresholds [13]. Esters have fruity notes and are mainly found in Italian type cheese, while methyl ketones are associated with fruity, musty and blue cheese notes and are typically found in mould-ripened cheese.

Thirteen (13) compounds were found only with milk as medium of which 7 compounds were found only in the ripened cheese. These include alcohols (4-methyl-1-heptanol, 6-methyl-1-heptanol, 3-ethyl-4-methylpentanol, 2-propanol, 2-butanol, 2-pentanol and 2-heptanol) and ketones (2-octanone, 6-methyl-5-hepten-2-one and δ-nonalactone) that most likely originate from degradation of milk fats.

In total 27 compounds were identified that were not found in the 4- or 8-week-old milli-cheeses, of which 24 were found in the retentostat cultures including 17 compounds that can be found in ripened cheese [13–18]. These 24 compounds include 2 diketones with a buttery, cheesy flavour (2,3-pentanedione and 2,3-heptanedione) [19], 3 unsaturated ketones with a sweet, cheesy flavour (3-buten-2-one, 3-penten-2-one and 3-hepten-2-one) [20], 4 unsaturated short aldehydes with a green fruity aroma (2-butenal, 3-methyl-2-butenal, 2-methyl-2-butenal and 2-ethyl-2-butenal) [21] and 7 long straight-chain alcohols, ketones and aldehydes that have a relatively high perception threshold and are conceivably less important (1-dodecanol, 1-tetradecanol, 1-hexadecanol, 2-tridecanone, 2-pentadecanone, tridecanal and pentadecanal). Based on these odour descriptions, production of these compounds is considered positive.

**Fig. 2:**
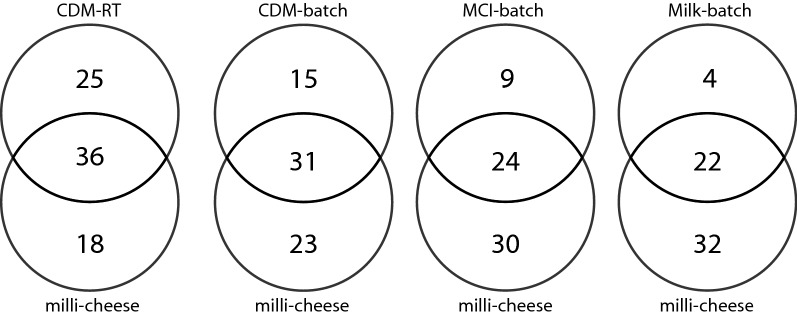
Qualitative comparison of the production of VOCs by *L. lactis* FM03-V1. VOCs production was compared between the liquid cultures and a milli-cheese model system after 8 weeks of ripening

Comparing all produced aroma compounds with ripened milli-cheeses revealed that the aroma profile of the retentostat cultures had the biggest overlap with 8-week-old milli-cheeses (36 compounds in common) despite the use of a chemically defined medium (Fig. 2). Moreover, many of the aroma compounds that were produced in the retentostat cultures but not in the milli-cheeses can be found in other types of cheeses [13–18] and/or have an odour description that is considered positive. This shows that retentostat cultivation is an interesting technique to produce aroma compounds typical for cheese.

### Quantitative comparison presence of aroma

**Fig. 3:**
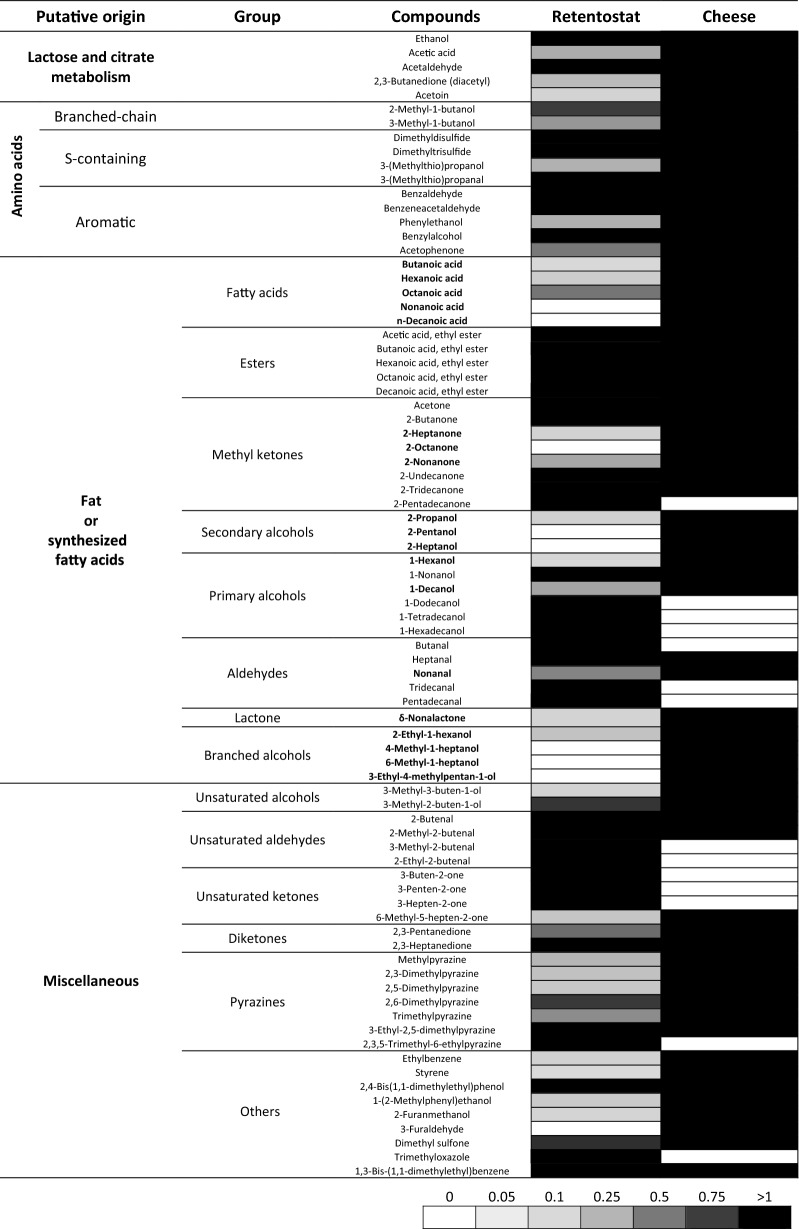
Quantitative comparison of VOCs in retentostat cultures of *L. lactis* FM03-V1 with a milli-cheese model system. Shades of grey correspond to the abundance relative to the 8-week-old milli-cheese (see legend at bottom of table). Black: similar or higher than in milli-cheese; grey: lower than in milli-cheese; white: absent. Production of compounds in bold was lower in the retentostat cultures but might increase by addition of free fatty acids to the medium

To identify compounds that were underrepresented in the retentostat cultures, we quantitatively compared aroma formation in the retentostat cultures with that in the 8-week-old cheese. We combined this information with the putative origin of the compounds indicating which substrates could be added to the medium to enhance flavour formation during retentostat cultivation to better resemble aroma formation during cheese ripening (Fig. 3). In total 25 out of 67 compounds found in the 8-week-old milli-cheese model system were found in higher amounts in the retentostat culture and 33 compounds were produced in the retentostat cultures but in lower amounts. Only 9 compounds were lacking in the retentostat cultures (peak area in retentostat culture < 1% of milli-cheese).

Groups of compounds that were underrepresented in the retentostat cultures were mainly medium-chain free fatty acids, medium-chain methyl ketones, secondary alcohols, medium-chain primary alcohols and branched-chain primary alcohols (Fig. 3). These compounds most likely originate from the milk fats via lipolysis and subsequent conversion of the released free fatty acids to either methyl ketones and secondary alcohols via β-oxidation or primary alcohols and branched-chain alcohols via reduction of the free fatty acids. Fat or free fatty acids were not present in the chemically defined medium and therefore the fatty acids that were found were synthesised by the bacteria. Fatty acids biosynthesis involves the addition of two carbon units in each cycle explaining why only even numbered fatty acids were found in the retentostat cultures. During β-oxidation, fatty acids are first oxidised to α-ketoacids, which are further decarboxylated to their corresponding methyl ketones with one carbon atom less, such as 2-heptanone, 2-nonanone and 2-undecanone. Ultimately, the methyl ketones can be reduced to secondary alcohols [22]. Fatty acid biosynthesis in bacteria is regulated by product inhibition by long-chain acyl-ACPs [23] preventing high levels of fatty acid production. Therefore, it will be hard to find conditions to stimulate fatty acid production and it is more convenient to add fat or a mixture of free fatty acids to improve the production of fatty acid-related aroma compounds. Moreover, this enables the production of even numbered methyl ketones that are currently lacking in the retentostat cultures. Because fat will most likely impair the retentostat cultivation by clogging the filter, free fatty acids are preferred. Care should be taken with the addition of free fatty acids as high levels can lead to rancidity and unbalanced flavours [22]. As fatty acids and their derived compounds were also lacking after fermentation of MCI (Additional file 1: Figure S2), addition of free fatty acids could also improve flavour formation in MCI.

## Conclusions

Aroma production by *L. lactis* was clearly affected by the type of medium (CDM, MCI or milk) and the cultivation method (batch, retentostat or milli-cheese). Aroma formation in a milli-cheese model system was best resembled using retentostat cultivation in which the bacteria hardly grew mimicking cheese ripening conditions. Retentostat cultivation resulted in the highest number of known cheese aroma compounds (52) and increase in esters, methyl ketones, diketones and unsaturated ketones, which are important cheese flavours and/or have cheese-like or buttery flavours. In the retentostat cultures as well as in batch cultures with CDM and MCI, free fatty acids and their corresponding degradation products (methyl ketones, secondary alcohols and primary alcohols) were underrepresented compared to the milli-cheese. Addition of a mixture of free fatty acids to CDM and MCI could help to enhance flavour formation in these media to better resemble flavour formation in cheese. This study shows that typical cheese aroma compounds can be produced by lactic acid bacteria outside the cheese matrix, which offers opportunities for future applications as food supplements in dairy or non-dairy products.

## Additional file

**Additional file 1: Figure S1.** Biomass accumulation of *L. lactis* FM03-V1 in retentostat cultures. **Figure S2.** Quantitative comparison of the volatile organic acids in liquid cultures of *L. lactis* FM03-V1 and in a milli-cheese model system.
