# Supplementary material for: Aroma formation during cheese ripening is best resembled by Lactococcus lactis retentostat cultures
Source: Microb Cell Fact. 2018 Jul 4;17:104. doi: 10.1186/s12934-018-0950-7 (PMC6030761; doi:10.1186/s12934-018-0950-7)
Supplement: Supplementary file 1 — Additional file 1: Figure S1. Biomass accumulation of L. lactis FM03-V1 in retentostat cultures. Figure S2. Quantitative comparison of the volatile organic acids in liquid cultures of L. lactis FM03-V1 and in a milli-cheese model system. [file 12934_2018_950_MOESM1_ESM.pdf]

Additional file 1

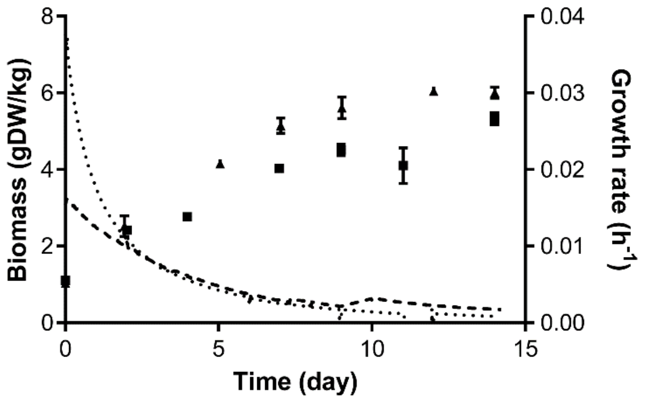

Figure S1: Biomass accumulation of *L. lactis* FM03-V1 in two independent retentostat cultures at a dilution rate of 0.05 h^{-1} and the corresponding growth rates. Squares and triangles represent biomass concentrations of retentostat 1 and 2, respectively. Dashed and dotted lines represent the estimated growth rates in retentostat culture 1 and 2, respectively. Error bars represent the standard deviation of technical duplicates.

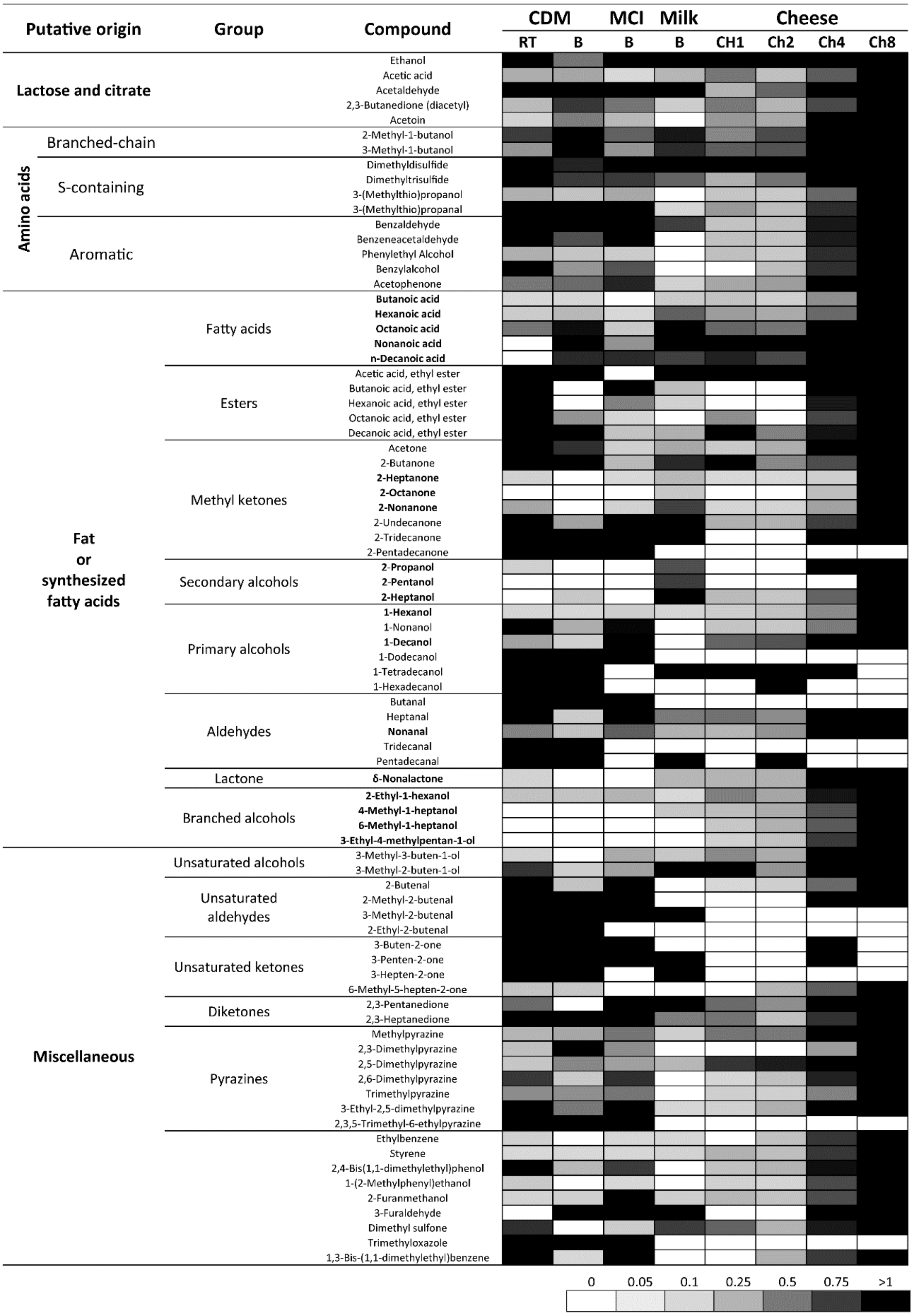

Figure S2: Quantitative comparison of the volatile organic compounds in liquid cultures of *L. lactis* FM03-V1 and in a milli-cheese model system after 1, 2, 4 and 8 weeks of ripening. Shades of grey correspond to the abundance relative to the 8-week-old milli-cheese (see legend at bottom of table). Black: similar or higher than milli-cheese; grey: lower than in milli-cheese; white: absent. B: batch cultivation; RT: retentostat cultivation, CDM: chemically-defined medium; MCI: hydrolysed micellar casein isolate medium.
